# Supplementary material for: Factors associated with Covid-19 vaccine acceptance among persons with disabilities: A cross-sectional study in Ghana
Source: PLOS Glob Public Health. 2024 Mar 14;4(3):e0002822. doi: 10.1371/journal.pgph.0002822 (PMC10939259; doi:10.1371/journal.pgph.0002822)
Supplement: Attachment — Submitted filename: Response to reviewers.docx [file pgph.0002822.s003.docx]

Godfred Atta-Osei	22^nd^ November, 2023.

Kwame Nkrumah University of Science and Technology.

School of Public Health

Department of Health Promotion and Disability Studies

THE EDITOR

PLOS GLOBAL PUBLIC HEALTH

Dear Sir/Madam

We would like to thank you for the opportunity to resubmit a revised copy of our manuscript and to thank you for the extension of revision deadline. We would also like to express our thanks to the reviewers for their positive feedback and helpful comments which would improve the quality of our manuscript.

The manuscript has been revised to address comments raised by the reviewers appended to this document. We believe that this revised manuscript would be accepted for publication in the Plos Global Public Health Journal.

Sincerely yours,

Godfred Atta-Osei

On behalf of all authors

|  | **Reviewer 1** | |
| --- | --- | --- |
|  | Comment | Response |
| 1. | Line 15-16: (research on Covid-19 vaccine acceptance has not targeted PwDs) -Before you write this, you should state whether there has been research among other groups. Also the justification of the study should be clearly stated…That is, the rationale of this study | While COVID-19 has had a wide-ranging impact on individuals and societies, persons with disabilities are uniquely affected largely due to secondary health conditions and challenges in adhering to protective measures. However, research on COVID-19 and vaccine acceptance has primarily focused on the general population and healthcare workers but has specifically not targeted PwDs, who are more vulnerable within societies. Hence, this study assessed PWDs knowledge of COVID-19 and factors associated with COVID-19 vaccine acceptance. |
| 2. | Line 18 - 21: Try to rewrite this section. It is not comprehensive enough, and the method should include; study design, study population, sampling techniques, data collection (you can include the questionnaire/research tool used), and data analysis | A cross-sectional survey was conducted among PWDs in the Atwima Mponua District in the Ashanti Region of Ghana. Respondents were sampled systematically and data was collected using a structured questionnaire. The data were analyzed with STATA version 16.0. Descriptive analysis was done using means and proportions. The chi-square test and Logistic regression were used to assess Covid-19 vaccine acceptance among the respondents. |
| 3. | Line 22 - 31: Start the result section with the descriptive analysis (like mean age, age range, etc) before proceeding to the inferential analysis result | 250 PWDs were recruited for the study. The minimum age of respondents was 18 and the maximum age was 90. Their average was found to be 50.2 with a standard deviation of 19.5.  A higher proportion of the respondents were females, physically impaired, and between 30 – 50 years. The majority (74%) of the PWDs had average knowledge about Covid-19. Factors such as age, educational level and type of disability were significantly associated with PWDs’ knowledge of COVID-19. The acceptance rate for COVID-19 among PwDs was 71.2%. Age, religion, knowledge of COVID-19, and educational level were significantly associated with Covid-19 vaccine acceptance. Persons with disabilities with low and average knowledge of COVID-19 were 95% and 65%, respectively, less likely to accept the vaccine compared to those with high knowledge of COVID-19 (AOR=0.05, 95%CI: 0.01, 0.21; AOR=0.35, 95%CI: 0.12, 1.03). Older people and those with higher education were more likely to accept the vaccine compared to younger people and those with no or less education. |
| 4. | Line 31 - 35 The sentence is too long, and looks confusing…try to rephrase it. | Persons with disabilities have average knowledge of COVID-19 and a greater percentage of them were willing to accept the vaccine. The study identified age, religion, knowledge of COVID-19, and educational level as contributing factors to their willingness to accept the COVID-19 vaccine. This suggest that PWDs will lean positive toward COVID-19 vaccine programs and as such, vaccination programs should target them. |
| 5. | Line 46: Be consistent. Are you using the term ‘coronavirus” or ‘Covid-19’...Stick with one | Coronavirus is replaced with Covid-19 |
|  | Line 109 - 110 You should not mention that the questionnaire was made up of six sections since this study only focused on two sections. You can just state that the questionnaire used in this study was made up of two sections  Line 112 - 113 But why is section two measuring both the independent and dependent variables…Each section should stand for each variable | Both comments are address as follows:  A structured questionnaire made up of three sections was administered to respondents. Section one comprised respondents’ socio-demographic characteristics such as age, gender, impairment type, level of education, marital status, employment status, level of income, and religious background. Section two featured the knowledge of Covid-19. Section three which was only one statement, measured whether or not PwDs will accept Covid-19 Vaccine. The data were collected between August and September 2021. Three members of the research team comprising two with expertise in Ghanaian Sign Language Interpretation administered the questionnaires to respondents in their communities. Some respondents opted to complete the questionnaires themselves with guidance from the researchers. For those who could not complete the questionnaire on their own, the researchers read and explained each statement on the questionnaire in Asante Twi language to their understanding before making a choice. The researchers signed statements on the questionnaire in Ghanaian sign language to the understanding of deaf participants. They were allowed some time to make their choice before moving to the next statement |
| 6. | Line 131 - 133: Start with the mean age, then age range, and others | The below have been added to the write-up  The minimum age of respondents was 18 and the maximum age was 90. Their average was found to be 50.2 with a standard deviation of 19.5. |
| 7. | Line 141: The percentage should be in one decimal place | Addressed |
| 8. | Line 141 -158: Try to rephrase the write-up. Also, be consistent with the flow of table. Do not back and forth on the table, report the result chronologically, as it appears in the table  Line 147: (15%) - State the exact value. 14.8% not 15%; condition - What condition? Try to state it; the average correct response was 70% - Where did this come from  Line 153: 90% - Use the exact value…do not round up | All comments have been addressed as follows:  Table 2 presents the knowledge of Respondents on Covid-19 outbreak. Generally, study observed that respondents have good knowledge of Covid-19. About 14.8 respondents had high knowledge of the Covid-19 and majority (74.0%) of the respondents had average knowledge of Covid-19. For example, most (88%) respondents stated that Covid-19 is a viral infection, while a few stated otherwise. About 86.4% of the respondents indicated that Covid-19 is transmitted by close contact with an infected person. Regarding Covid-19 being declared as epidemic, 170 respondents representing 68%, responded no, meanwhile Covid-19 is declared as a pandemic. A percentage majority (75.6%) of the respondents indicated that the incubation period of Covid-19 is one month, which is not true and a little above half of the respondent (54.8%) indicated that Covid-19 is curable. On the transmission of the virus, 89.6 stated that visiting crowded places and being close to people showing symptoms increase ones’ risk of Covid-19 infection and handwashing with soap was indicated by the majority (90.8) as a safety protocol to prevent the spread of the disease. |
| 9. | Line 160 - 172: For this section, you do not need to write about the percentage. You should only be concerned with the p-value and association  Line 161: Analysis - State the analysis used  Line 166 - 167: The type of disability was also found to have statistical significance on respondent’s level of knowledge on Covid-19 (p=0.055) - It was not significant. 0.055 is not significant. | Addressed |
| 10. | Line 177 - 181: You do not need to write everything from the table; but if you want to write it, you can try to put the sentences together as one e.g PwDs aged 36-50 years and 51-65 years are 3.1 times and 3.7 times respectively more likely to accept Covid-19 vaccine compared to those between 20-35 years (COR=3.08, 95%CI=1.44-6.61; COR=3.70, 95%CI=1.55-8.82 respectively). | PwDs aged 36-50 years and 51-65 years are 3.1 times and 3.7 times respectively more likely to accept Covid-19 vaccine compared to those between 20-35 years (COR=3.08, 95%CI=1.44-6.61; COR=3.70, 95%CI=1.55-8.82 respectively).  Has been incorporated |
| 11. | Line 209 - 217: This is not necessary…It should be removed | Removed |
| 12. | Line 277 - 278: There is a section of persons with disabilities who have not registered with the department - Rephrase this | The sentence is removed and rephrased in the next sentence as:  This means that the views of PwDs in the district who have not registered with department of social welfare were not captured in the current study. |
|  | **Reviewer 2** | |
| 1. | Reviewer #2: The authors may consider revising sentence on page 20 line 213 as follows: "Measures taken against the spread of Covid-19 virus" | This portion has been removed following the advice of reviewer 1 |
| 2. | Also a clear description of method employed in interviewing persons with hearing impairment could clarify the results as presented | Addressed |
