# Supplementary material for: Factors associated with Covid-19 vaccine acceptance among persons with disabilities: A cross-sectional study in Ghana
Source: PLOS Glob Public Health. 2024 Mar 14;4(3):e0002822. doi: 10.1371/journal.pgph.0002822 (PMC10939259; doi:10.1371/journal.pgph.0002822)
Supplement: Attachment — Submitted filename: Response to editor.docx [file pgph.0002822.s004.docx]

Godfred Atta-Osei	5^th^ February, 2024.

Kwame Nkrumah University of Science and Technology.

School of Public Health

Department of Health Promotion and Disability Studies

THE EDITOR

PLOS GLOBAL PUBLIC HEALTH

Dear Editor

We would like to thank you for the opportunity to resubmit a revised version of our manuscript. We appreciate your positive feedback and helpful comments which would improve the quality of our manuscript.

The manuscript has been revised to address the comments you raised and appended to this document. We believe that this revised manuscript would be accepted for publication in the Plos Global Public Health Journal.

Sincerely yours,

Godfred Atta-Osei

On behalf of all authors

|  | **Editor** | |
| --- | --- | --- |
|  | Comment | Response |
| 1. | Table Four | We observed that the table with P-Values and Confidence intervals is table five rather. |
| 2. | Can you be consistent in your use of PWD vs PwD (I think just abstract has PWD)? | All PWDs appeared in the manuscript has been changed to PwDs |
| 3. | In Table 4 can you make a note of what variables are included in the adjusted model? (Even if it's a footnote saying something like: "Adjusted analysis includes all covariates listed in this table.") | Comment 3 has been addressed: see line 387  “**Note**: The adjusted model included all variables in the table.”  We have provided another further information in the statistical analysis. Find below in Line 142-144  “Also, variables of significant importance from other studies on vaccine acceptance that were not” significant in the univariate analysis were accounted for in the multivariate model. |
| 4. | In Table 4, could you switch order of confidence intervals and P-values? | Order of confidence intervals and P-Values have been changed accordingly. |
| 5. | I will note that stepwise selection procedures are not very recommended anymore. If you want to keep it in, that is fine, but a better approach may be to a priori decide on a list of covariates to include in the model, and to just adjust for all of those, regardless of bivariate significance. | We would like to maintain the stepwise selection procedure at this time. We hope to observe use a different approach in our subsequent manuscripts. |
